# Clinical landscape for patients with head and neck cancers enrolled in phase I trials at a tertiary referral center

**DOI:** 10.1177/17588359251337244

**Published:** 2025-06-30

**Authors:** Daria Maria Filippini, Raphael Sanchez, Etienne Bastien, Nicolas Jacquin, Alexandro Paccapelo, Caroline Nhy, Jerzy Klijanienko, Valentin Calugaru, Anne Chilles, Wahib Ghanem, Guillaume Rougier, Antoine Dubray Vautrin, Nathalie Badois, Maria Lesnik, Olivier Choussy, Marie Paule Sablin, Christophe Le Tourneau, Grégoire Marret

**Affiliations:** Department of Experimental, Diagnostic and Specialty Medicine, University of Bologna, Sant’ Orsola-Malpighi Hospital, Pietro Albertoni Street, Bologna I-40138, Italy; Department of Drug Development and Innovation (D3i), Institut Curie, Paris, France; Department of Drug Development and Innovation (D3i), Institut Curie, Paris, France; Department of Drug Development and Innovation (D3i), Institut Curie, Paris, France; Research and Innovation Unit, IRCCS Azienda Ospedaliero-Universitaria di Bologna, Bologna, Italy; Radiology Department, Institut Curie Hospital, Paris, France; Department of Pathology, Institut Curie, PSL Research University, Paris, France; Department of Radiation Oncology, Institut Curie, Paris, France; Department of Radiation Oncology, Institut Curie, Paris, France; Department of Head and Neck Surgery, Institut Curie, Paris, France; Department of Head and Neck Surgery, Institut Curie, Paris, France; Department of Head and Neck Surgery, Institut Curie, Paris, France; Department of Head and Neck Surgery, Institut Curie, Paris, France; Department of Head and Neck Surgery, Institut Curie, Paris, France; Department of Head and Neck Surgery, Institut Curie, Paris, France; Department of Drug Development and Innovation (D3i), Institut Curie, Paris, France; Department of Drug Development and Innovation (D3i), Institut Curie, Paris, France; INSERM U900 Research Unit, Saint-Cloud, France; Paris-Saclay University, Paris, France; Department of Drug Development and Innovation (D3i), Institut Curie, Paris, France

**Keywords:** biomarkers, clinical drug development, clinical trials, drug development, head and neck cancer, outcomes research, phase I trials, prognostic biomarkers

## Abstract

**Background:**

Recent advances in understanding the biology of cancer have resulted in an extensive armamentarium of new therapeutic agents, most often tested on various tumor types at the earliest stages of drug development. However, the clinical impact of these therapies on patients with head and neck cancer (HNC) remains underexplored and requires further evaluation.

**Objectives:**

To investigate the clinical outcomes and toxicity profiles of patients with HNC enrolled in phase I trials (Ph1t) at a tertiary referral center over the last decade.

**Design:**

A retrospective cohort study was conducted, analyzing data from HNC patients enrolled in phase I trials at the Curie Institute between October 2011 and January 2024.

**Methods:**

Data on baseline characteristics, hematologic biomarkers, and outcomes were extracted from medical records. Objective response rate (ORR) and Kaplan–Meier estimates of progression-free survival (PFS) and overall survival (OS) were analyzed. A Cox model was used for the identification of prognostic factors.

**Results:**

One hundred and thirty patients were enrolled in Ph1t for recurrent/metastatic (R/M) setting (66.9%), including 20.8% of patients being treated with more than two lines of therapy, followed by locally advanced (LA) treated with radical surgery or exclusive chemo/radiotherapy (17.7%), neoadjuvant (10.0%), and adjuvant (5.4%) Ph1t. Patients were treated with immunotherapy (53.8%), targeted therapy (23.1%), bispecific antibody (8.5%), antibody–drug conjugate (4.6%), and other agents (10.0%). In 122 patients evaluable for response, ORR were 16.5%, 87.0%, and 92.3% in R/M, LA, and neoadjuvant Ph1t, respectively. Median PFS/OS rates were 2.0/8.3, 21.5/38.3, and 20.0/27.4 months in R/M, LA, and neoadjuvant Ph1t, respectively.

At multivariable analysis, lower lymphocytes (HR = 0.144; 95% CI: 0.052–0.399; *p* < 0.001) and lower albumin levels (HR = 0.922; 95% CI: 0.879–0.966; *p* < 0.001) remained associated with poorer OS. Grade 3–4 adverse events were recorded in 27/130 patients (20.8%). The most frequent were hematologic and gastrointestinal disorders. No treatment-related deaths occurred.

**Conclusion:**

HNC Ph1t show encouraging results in terms of early efficacy signals and safety profiles, emphasizing their value across a variety of clinical settings.

## Introduction

Head and neck squamous cell carcinoma (HNSCC) is the seventh most common cancer worldwide, with over 800,000 incident cases and 400,000 deaths in 2020. The overall incidence of HNSCC is expected to rise over the next 30 years, with a decrease in the incidence of tobacco-induced cancers and an increase in human papillomavirus (HPV)-induced cancer.^1^

Most of patients present with locally advanced (LA) disease at diagnosis and about 10%–20% with distant metastases. Despite advancements in surgical management, radiotherapy, and systemic treatments, relapse rates remain high, with approximately 50% experiencing recurrence due to inherent or acquired resistance mechanisms, underscoring the need for novel therapeutic strategies.

Treatment landscape is changed over the last years due to the introduction of immune checkpoint inhibitors (ICIs) for the recurrent/metastatic (R/M) setting but their benefits are limited to a minority of patients, with response rates of 20%–30% and median overall survival (OS) of approximately 11–15 months.^2,3^

Head and neck cancer (HNC) poses a significant challenge in drug development due to their aggressive behavior, molecular heterogeneity, and the scarcity of approved therapies.^4–6^ In this context, phase I clinical trials are necessary to develop precision medicine in HNC by providing a better understanding of the molecular landscape and insights into the feasibility, safety, and preliminary efficacy of experimental treatments.^7^

Phase I clinical trials are the first step in evaluating a new drug in humans. These trials primarily focus on assessing the safety profile of a treatment, determining the appropriate dose level. The key objective is to establish the maximum tolerated dose and understand how the drug behaves in the body, which includes studying its pharmacokinetics—the way the drug is absorbed, distributed, metabolized, and eliminated. Phase I trials typically involve a small group of patients, and the data gathered during this phase are crucial for guiding subsequent phases of clinical development. These trials are essential for testing new experimental treatments and advancing precision medicine.^8^

Moreover, for HNC, these trials could not only contribute to the advancement of cancer therapeutics but also offer the opportunity to access innovative interventions that may improve their prognosis, especially given the limited standard treatment options.

However, it has been observed that the predominant management of HNC by surgeons and radiation oncologists may inadvertently reduce the focus on chemotherapeutic trials, particularly in the early treatment settings.

Historical studies showed very low response rates in phase I trials across various cohorts of solid tumors, around 5%, prompting ethical concerns.^9^ Recent advances, including novel immunotherapy and targeted therapies, have significantly improved this response rate to around 20%.^10^

Whether these advancements extend to HNC patients, given their unique tumor biology and clinical challenges, remains an open question.

This retrospective study aims to evaluate clinical outcomes for HNC patients enrolled in phase I trials at a tertiary referral center during the last 10 years. By exploring patient and tumor characteristics, treatment efficacy, and safety profiles, we seek to elucidate the potential of phase I trials in fulfilling the therapeutic requirements of this challenging population, when no effective standard treatments are available, or in offering an opportunity in earlier treatment settings.

## Patients and methods

### Study population

We conducted a retrospective study of phase I clinical trials involving patients with HNC at the “Drug Development and Innovation” of the Curie Institute in Paris, France, from October 2011 to January 2024. All patients affected by HNC in the neoadjuvant, adjuvant, LA, and R/M settings who received at least one dose of experimental treatment were included. Patients who failed screening and did not receive treatment were excluded.

Each study received approval from an international review board and a local ethics committee. All patients provided written informed consent for their participation in phase I trials and for the use of their data for research purposes.

### Data collection

We collected patient data from electronic medical records. We extracted patient demographic characteristics, tumor histology and its relation with p16/HPV, hematologic parameters, various drug classes employed in phase I clinical trials, the number of previous therapy lines, reasons for trial discontinuation, grade (G) 3 or higher treatment-related adverse events (TRAEs) according to the common terminology criteria for adverse events (CTCAE) version 5.0,^11^ best response obtained within the trial, the presence of disease recurrence/progression, and living status (dead or alive). Patient characteristics included sex, age, performance status (PS) measured by the Eastern Cooperative Oncology Group (ECOG) PS, tumor histology, site and subsite, status of HPV if known through p16 as a surrogate marker of the HPV presence detected in immunohistochemistry (IHC) or/and through HPV-DNA detected in polymerase chain reaction (PCR), date of disease diagnosis, duration from the end of curative treatment to the first recurrence not amenable to surgery or radiotherapy/re-irradiation, prior lines of medical therapy in the R/M setting, presence of liver and lung metastases, disease setting at the start of experimental treatment (neoadjuvant, adjuvant, LA, R/M), experimental class of drugs, start/end date of experimental treatment, and reason for treatment discontinuation. In addition, from hematologic analyses, we collected data on baseline leukocytes, neutrophil-to-lymphocyte ratio,^12^ platelets, albumin, and lactate dehydrogenase (LDH). Disease response was assessed using the response evaluation criteria in solid tumors (RECIST) version 1.1.^13^ RECIST assessments are performed according to the evaluation windows specified in each protocol.

Patients were enrolled in phase I trials evaluating different classes of experimental drugs: bispecific antibody, antibody–drug conjugate, targeted therapy, immunotherapy alone or in combination with chemotherapy, with targeted therapy, targeted therapy combined with chemotherapy, and “other” treatments (referred to as a radiosensitizer). A list of trials included is reported in Supplemental Table 2.

We also analyzed patient safety in each disease setting, reporting the rate of G3, 4 or 5 TRAEs according to CTCAE 5.0 guidelines, and one patient may have had several TRAEs.

The reporting of this study conforms to the STROBE guidelines (Supplemental Table 3).^14^

### Statistical analysis

Patient demographic and clinical characteristics are reported as frequencies and percentages for categorical variables and as mean ± standard deviation or median and interquartile range (IQR) for continuous variables. Objective response rate (ORR) was defined as the proportion of patients who had either a partial response (PR) or a complete response (CR) to therapy. Disease control rate (DCR) was defined as the proportion of patients with CR, PR, or stable disease (SD).

The Chi-square test, Fisher’s exact test, the Kruskal–Wallis test, and the Mann–Whitney *U* test were used to analyze homogeneity between groups.

Progression-free survival (PFS) was defined as the duration from the first study treatment to disease progression or death from any cause. OS was defined as the time from the start of treatment to death for any reason. PFS and OS were estimated by the Kaplan–Meier method.

The Cox proportional hazards model was used for univariable and multivariable survival models. The hazard ratios (HRs) were computed together with their 95% confidence intervals (95% CIs). Non-normally distributed variables were normalized with the logarithmic function.

All statistical tests were two-sided, and *p* < 0.05 was considered statistically significant.

Statistical analyses for efficacy were performed using Stata version 18.0.^15^

## Results

### The flow chart diagram and baseline patients’ characteristics

**Figure 1.**
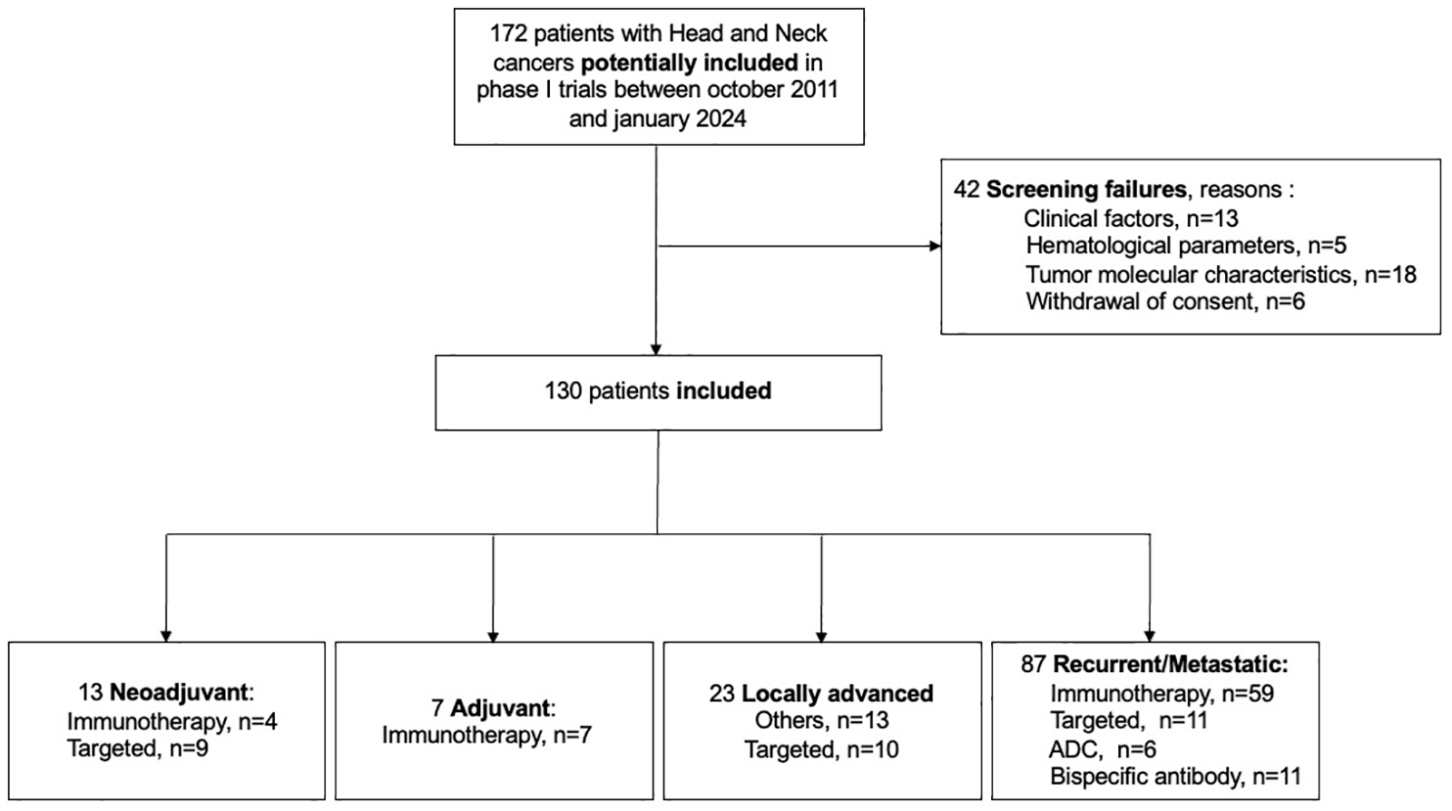
Flowchart of patients’ inclusion in phase I trials. Of the 172 patients with head and neck cancers screened between October 2011 and January 2024, 130 were included.

The patients’ selection flowchart is shown in Figure 1.

Among 172 patients initially assessed for phase I trials between October 2011 and January 2024, 42 were excluded due to screening failures, and 130 were included in the final analysis. These patients come from four settings: 13 (10.0%) from the neoadjuvant setting, 7 (5.4%) from the adjuvant setting, 23 (17.7%) from the LA setting, and 87 (66.9%) from the R/M setting.

The baseline characteristics of patients categorized into different settings are reported in Table 1.

**Table 1.** Baseline patients’ characteristics and outcomes based on disease setting.

Characteristics	Disease setting at experimental treatment start						Non recurrent	*p*-Value versus recurrent
	Neoadjuvant	Adjuvant	Locally advanced	Recurrent	Total	*p*-value		
*N*	13 (10.0%)	7 (5.4%)	23 (17.7%)	87 (66.9%)	130 (100.0%)		43 (33.1%)	
Age at treatment start (IQR)	64 (53–72)	59 (56–65)	73 (61–78)	60 (54–65)	61.500 (55–69)	<0.001	66 (56–76)	0.001
Male sex	11 (84.6%)	7 (100.0%)	19 (82.6%)	75 (86.2%)	112 (86.2%)	0.707	37 (86.0%)	1.000
ECOG PS								
0	8 (61.5%)	6 (85.7%)	10 (43.5%)	25 (28.7%)	49 (37.7%)	0.004	24 (55.8%)	0.004
⩾1	5 (38.5%)	1 (14.3%)	13 (56.5%)	62 (71.3%)	81 (62.3%)		19 (44.2%)	
Histology								
Squamous cell carcinoma	13 (100.0%)	7 (100.0%)	23 (100.0%)	79 (90.8%)	122 (93.8%)	0.648	43 (100.0%)	0.174
ACC	0 (0.0%)	0 (0.0%)	0 (0.0%)	5 (5.7%)	5 (3.8%)		0 (0.0%)	
Non-ACC (mucoepidermoid and acinic cell carcinoma)	0 (0.0%)	0 (0.0%)	0 (0.0%)	3 (3.4%)	3 (2.3%)		0 (0.0%)	
Subsite								
Oral cavity	1 (7.7%)	4 (57.1%)	2 (8.7%)	25 (28.7%)	32 (24.6%)	0.014	7 (16.3%)	0.004
Oropharynx	8 (61.5%)	2 (28.6%)	16 (69.6%)	30 (34.5%)	56 (43.1%)		26 (60.5%)	
Larynx/hypopharynx	4 (30.8%)	1 (14.3%)	5 (21.7%)	20 (23.0%)	30 (23.1%)		10 (23.3%)	
Others	0 (0.0%)	0 (0.0%)	0 (0.0%)	12 (13.8%)	12 (9.2%)		0 (0.0%)	
HPV								
Neg	2 (15.4%)	7 (100.0%)	5 (21.7%)	37 (42.5%)	51 (39.2%)	<0.001	14 (32.6%)	0.026
Pos	5 (38.5%)	0 (0.0%)	10 (43.5%)	12 (13.8%)	27 (20.8%)		15 (34.9%)	
NA	6 (46.2%)	0 (0.0%)	8 (34.8%)	38 (43.7%)	52 (40.0%)		14 (32.6%)	
Stage of experimental treatment start								
Locoregional disease	13 (100.0%)	7 (100.0%)	23 (100.0%)	0 (0.0%)	43 (33.1%)	<0.001	43 (100.0%)	<0.001
Locoregional recurrence only	0 (0.0%)	0 (0.0%)	0 (0.0%)	36 (41.4%)	36 (27.7%)		0 (0.0%)	
Metastatic only	0 (0.0%)	0 (0.0%)	0 (0.0%)	27 (31.0%)	27 (20.8%)		0 (0.0%)	
Both	0 (0.0%)	0 (0.0%)	0 (0.0%)	24 (27.6%)	24 (18.5%)		0 (0.0%)	
Prior lines of medical therapy								
⩾2 lines	13 (100.0%)	7 (100.0%)	23 (100.0%)	60 (69.0%)	103 (79.2%)	<0.001	43 (100.0%)	<0.001
>2 lines	0 (0.0%)	0 (0.0%)	0 (0.0%)	27 (31.0%)	27 (20.8%)		0 (0.0%)	
Lung metastases	0 (0.0%)	0 (0.0%)	0 (0.0%)	42 (48.3%)	42 (32.3%)		0 (0.0%)	<0.001
Liver metastases	0 (0.0%)	0 (0.0%)	0 (0.0%)	12 (13.8%)	12 (9.2%)		0 (0.0%)	0.008
Monotherapy	0 (0.0%)	4 (57.1%)	0 (0.0%)	33 (37.9%)	37 (28.5%)	<0.001	4 (9.3%)	<0.001
CLASS of experimental drug								
Immunotherapy	4 (30.8%)	7 (100.0%)	0 (0.0%)	59 (67.8%)	70 (53.8%)	<0.001	11 (25.6%)	<0.001
Targeted agent	9 (69.2%)	0 (0.0%)	10 (43.5%)	11 (12.6%)	30 (23.1%)		19 (44.2%)	
ADC	0 (0.0%)	0 (0.0%)	0 (0.0%)	6 (6.9%)	6 (4.6%)		0 (0.0%)	
Bispecific antibody	0 (0.0%)	0 (0.0%)	0 (0.0%)	11 (12.6%)	11 (8.5%)		0 (0.0%)	
Other	0 (0.0%)	0 (0.0%)	13 (56.5%)	0 (0.0%)	13 (10.0%)		13 (30.2%)	
Neutrophils, per dL	4.98 (3.19–6.69)	2.59 (2.35–6.33)	4.47 (3.23–7.58)	5.34 (3.73–7.74)	5.25 (3.58–7.61)	0.504	4.47 (3.17–7.09)	0.319
Leucocytes, per dL	6.70 (6.20–9.70)	3.93 (3.91–8.91)	7.00 (5.90–10.00)	7.19 (5.43–9.78)	7.17 (5.60–9.70)	0.501	6.90 (5.90–9.70)	0.870
Lymphocytes, per dL	1.90 (1.48–2.69)	0.83 (0.65–1.18)	1.66 (1.25–1.91)	0.93 (0.58–1.26)	1.11 (0.68–1.62)	<0.001	1.68 (1.18–2.05)	<0.001
dNLR	1.79 (1.67–2.49)	1.93 (1.51–2.45)	1.80 (1.57–2.44)	2.90 (2.03–4.40)	2.49 (1.77–4.00)	0.002	1.80 (1.57–2.45)	<0.001
Monocytes	612 (500–790)	520 (520–700)	670 (550–972)	660 (540–930)	655 (530–890)	0.589	640 (520–790)	0.462
Eosinophils	176 (110–249)	200 (130–590)	130 (102–280)	111 (60–220)	130 (100–230)	0.084	140 (110–253)	0.015
PLT, ×10^3^	242 (208–321)	191 (168–371)	284 (244–344)	271 (214–350)	272 (216–344)	0.528	279 (224–343)	0.835
Albumin	38 (38–45)	39.8 (39–40)	40 (38–42)	37 (34–40)	38 (35–41)	0.007	40 (38–42)	<0.001
LDH	360 (223–463)	161 (131–303)	186.500 (175–208)	215 (184–261)	208 (180–274)	0.002	202.500 (175–340)	0.550
Best response								
CR	4 (30.8%)	0 (0.0%)	10 (43.5%)	3 (3.8%)	17 (14.8%)	<0.001	14 (38.9%)	<0.001
PR	8 (61.5%)	0 (0.0%)	10 (43.5%)	10 (12.7%)	28 (24.3%)		18 (50.0%)	
SD	1 (7.7%)	0 (0.0%)	2 (8.7%)	20 (25.3%)	23 (20.0%)		3 (8.3%)	
PD	0 (0.0%)	0 (0.0%)	1 (4.3%)	46 (58.2%)	47 (40.9%)		1 (2.8%)	
Objective response								
No	1 (7.7%)	0 (0.0%)	3 (13.0%)	66 (83.5%)	70 (60.9%)	<0.001	4 (11.1%)	<0.001
Yes	12 (92.3%)	0 (0.0%)	20 (87.0%)	13 (16.5%)	45 (39.1%)		32 (88.9%)	
Treatment-related adverse events, grade ⩾3								
No	7 (53.8%)	7 (100.0%)	14 (60.9%)	74 (85.1%)	102 (78.5%)	0.005	28 (65.1%)	0.013
Yes	6 (46.2%)	0 (0.0%)	9 (39.1%)	13 (14.9%)	28 (21.5%)		15 (34.9%)	

The table also provides a comprehensive comparison between non-recurrent and recurrent cases. Statistical significance is assessed with *p*-values, with comparisons drawn both across disease settings and between recurrent and non-recurrent groups. Data are presented as frequencies and percentages for categorical variables and as median and IQR for continuous variables.

ACC, adenoid cystic carcinoma; ADC, antibody–drug conjugate; CR, complete response; dNLR, derived neutrophil-to-lymphocyte ratio; ECOG PS, Eastern Cooperative Oncology Group performance status; HPV, human papillomavirus; IQR, interquartile range; LDH, lactate dehydrogenase; *N*, number of patients; NA, not available; Objective response, combined measure of CR and PR; PD, progressive disease; PLT, platelets; PR, partial response; SD, stable disease.

Overall, the median age was 61 years (IQR: 55–69 years), with a majority of males (*n* = 112, 86.2%). Most patients had an ECOG PS of 0 or 1 (37.7% and 60.8%, respectively), with patients in the R/M setting having a worse PS (71.3% with ECOG ⩾1) compared to non-recurrent settings (a total of 44.2% with ECOG ⩾1) including those in the neoadjuvant (38.5%), adjuvant (14.3%), or suitable for surgery or chemo/radiotherapy in the LA (56.5%) settings (*p* = 0.004).

Nearly all patients had squamous histology (93.8%), whereas eight patients in the R/M setting with salivary gland cancers (five adenoid cystic carcinoma and three non-adenoid cystic carcinoma) were enrolled. The most common tumor site was the oropharynx (43.1%), followed by oral cavity (24.6%), larynx/hypopharynx (23.1%), and others (9.2%). The latter included nasopharyngeal cancers, salivary gland cancers, paranasal sinuses, and unknown primary of squamous cell carcinoma (SCC) of head and neck district.

HPV positivity was observed in 20.8% overall; however, in 40% of cases, the HPV status was unknown. Most of the patients (69.0%) in the R/M group had previously undergone two or fewer lines of therapy, and a minority were treated with more than two lines (31.0%).

According to the class of experimental treatment, immunotherapy was used in 53.8% of cases, predominantly in the adjuvant (100.0%) and R/M settings (67.8%), with statistically significant heterogeneity between groups (*p* < 0.001). Targeted agents accounted for 23.1% of treatments, more frequently in neoadjuvant and LA settings, while other categories, such as antibody–drug conjugate (4.6%), bispecific antibodies (8.5%), and “Other” (10.0%), were less frequently administered.

We detailed the list of experimental treatment classes and combinations, according to the number of trials and patients for each category, in Supplemental Table 1.

Regarding baseline biomarker analysis, neutrophil levels did not show any significant differences between the groups (*p* = 0.504). By contrast, lymphocyte counts displayed notable variation, with the adjuvant and R/M groups exhibiting significantly lower median values (0.83 and 0.93 per dL, respectively, *p* < 0.001). Similarly, the derived neutrophil-to-lymphocyte ratio (dNLR) differed significantly across the groups, with the highest level in the R/M group (*p* = 0.002). Albumin levels also showed significant variation with R/M showing the lowest value (37 g/L (range: 34–40)) (*p* = 0.007). Finally, LDH levels varied significantly with the adjuvant setting, showing the lowest value (*p* = 0.002), which may reflect differences in tumor burden or metabolic activity among the groups. Specifically, patients in the R/M group had elevated dNLR (2.9 vs 1.8; *p* < 0.001) and lower albumin levels (37 vs 40 g/L; *p* < 0.001) compared to non-recurrent groups (Table 1).

### Clinical outcomes across different settings

In terms of best response, CR was achieved by 14.8% of patients overall, with the highest rate seen in the LA group (43.5%), *p* < 0.001. A total of 24.3% of patients have obtained PR, with the neoadjuvant group showing a particularly high rate of 61.5%. SD was observed in 20.0% of patients, with the highest proportion occurring in the R/M group (25.3%). On the other hand, PD was noted in 40.9% of patients overall, with 58.2% of R/M patients, while one patient with LA disease experienced PD (Table 1).

**Figure 2.**
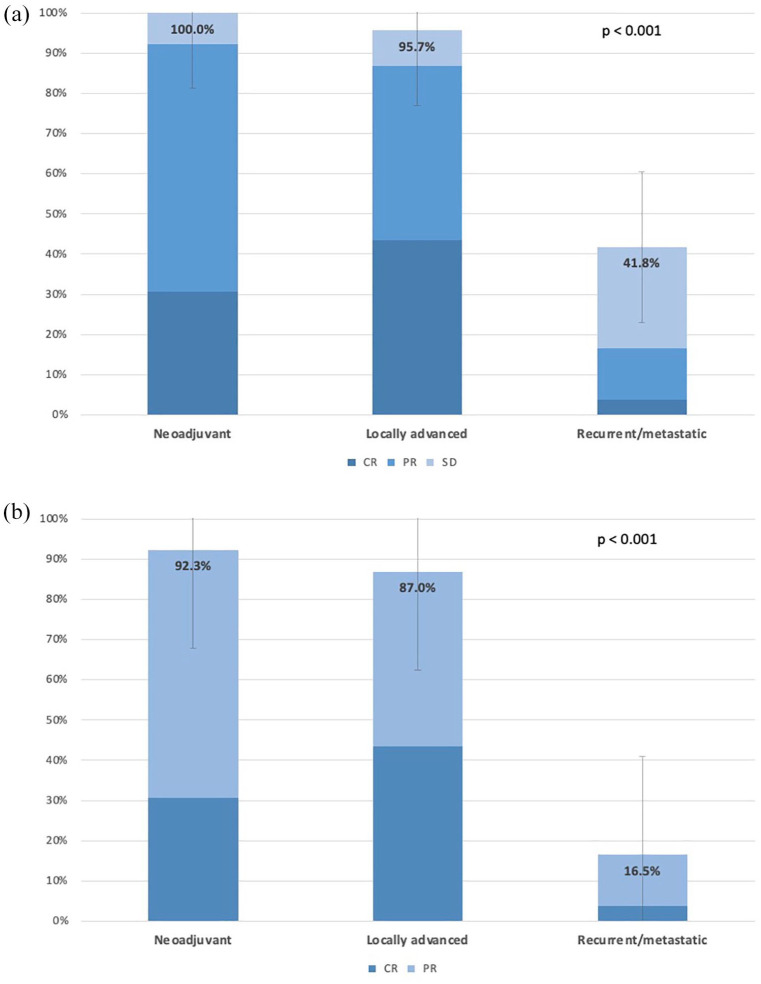
Disease control rate (a) and objective response rate (b) across different therapeutic settings, including neoadjuvant, locally advanced, and recurrent/metastatic. Radiological responses were assessed by RECIST 1.1 criteria. RECIST, response evaluation criteria in solid tumors.

Figure 2 reviews the DCR (Figure 2(a)) and ORR (Figure 2(b)) across different clinical settings.

In terms of DCR, the LA group shows a high DCR of 95.7%, while, as expected, the R/M group has a significantly lower DCR of 41.8% (*p* < 0.001). In the neoadjuvant setting, the DCR was 100%.

For the ORR, the neoadjuvant and LA setting demonstrate an ORR of 92.3% and 87.0%, respectively, compared to 16.5% in the R/M setting, *p* < 0.001.

**Figure 3.**
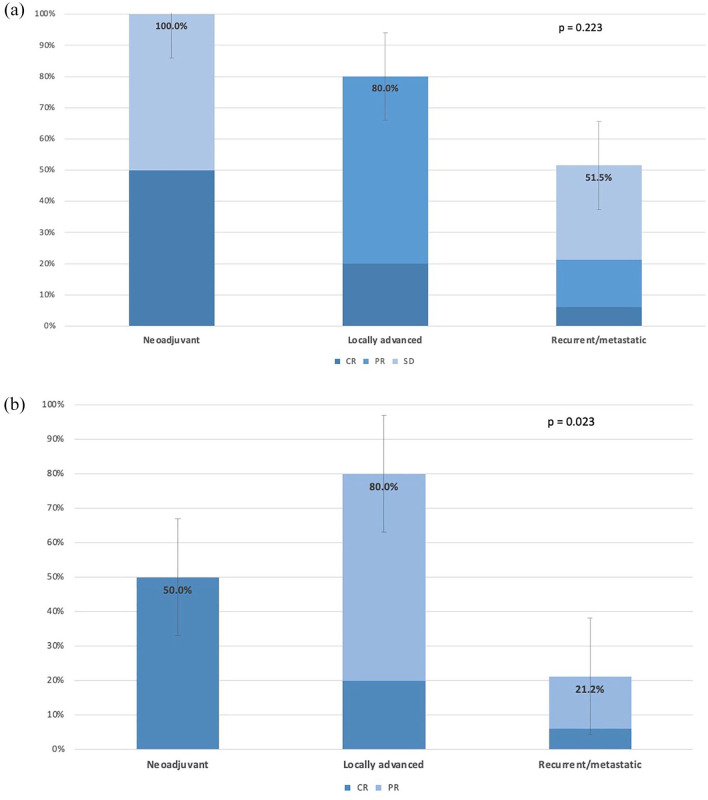
Disease control rate (a) and objective response rate (b) in HPV-negative tumors across different therapeutic settings, including neoadjuvant, locally advanced, and recurrent/metastatic. Radiological responses were assessed by RECIST 1.1 criteria. RECIST, response evaluation criteria in solid tumors.

For HPV-negative tumors, Figure 3(a) and (b) illustrates the DCR and ORR in this subset across different therapeutic settings, including neoadjuvant, LA, and R/M disease. The data reveal a DCR of 100% and an ORR of 92.3% in the neoadjuvant setting, a DCR of 95.7% and an ORR of 87.0% in the LA setting, and a DCR of 41.8% and an ORR of 16.5% in the R/M setting, respectively.

**Figure 4.**
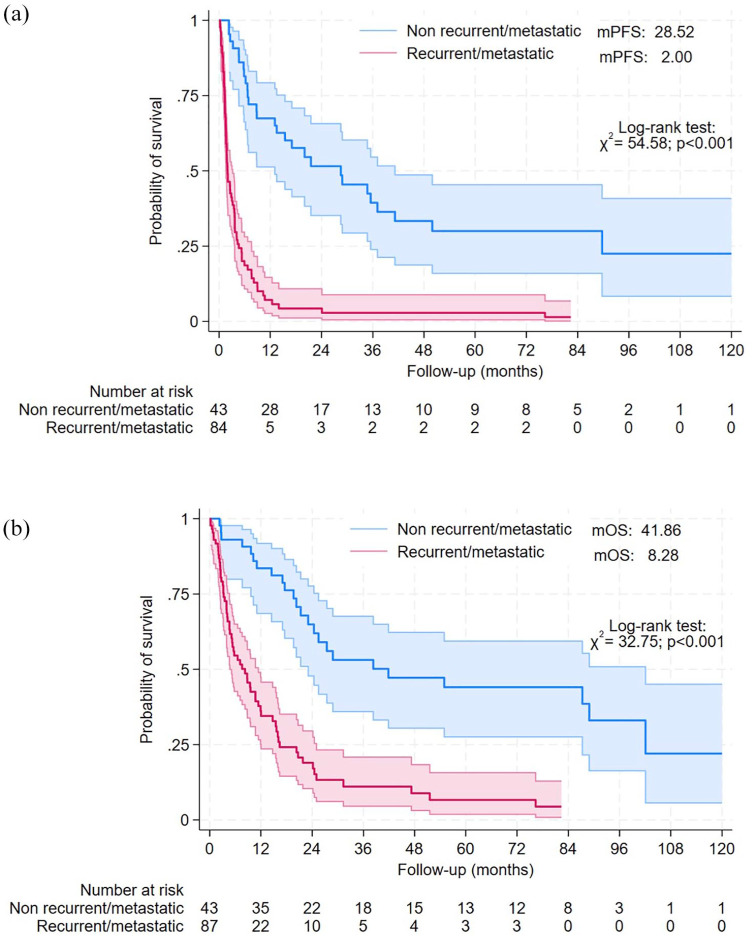
Survival analyses according to recurrent/metastatic and non-recurrent/metastatic groups. Progression-free survival (a) and overall survival (b) analyses.

The median PFS rates were 28.5 months (95% CI: 10.2–46.8 months) and 2.0 months (95% CI: 1.3–2.7 months) for the non-recurrent and R/M setting, respectively (*p* < 0.001) (Figure 4(a)).

The median OS rates were 41.9 months (95% CI: 4.5–79.2 months) and 8.3 months (95% CI: 4.6–12.0 months) for the non-recurrent and R/M setting, respectively (*p* < 0.001) (Figure 4(b)).

The PFS univariable analysis (Table 2) for prognostic factors at phase I trial enrollment, across all settings, revealed that prior lines of medical therapy >2 (HR = 2.730, *p* < 0.001), ECOG PS >0 (HR = 1.527, *p* = 0.044), lung metastases (HR = 2.180, *p* < 0.001), and liver metastases (HR = 2.489, *p* = 0.006) significantly increased the risk of PD; lymphocyte count was strongly protective, with a significant reduction in risk (HR = 0.121, *p* < 0.001), high dNLR was predictive of poor PFS (HR = 2.875, *p* = 0.010), and hypoalbuminemia was significantly associated with poorer outcomes (HR = 0.92, *p* < 0.001). In the multivariable analysis, prior lines of medical therapy >2 (HR = 2.007, *p* = 0.007), lymphocyte count (HR = 0.218, *p* = 0.001), and albumin levels (HR = 0.949, *p* = 0.013) remain significant.

**Table 2.** Univariable and multivariable analyses for progression-free survival and overall survival.

Characteristics	Progression-free survival (months)						Overall survival (months)					
	Univariable analysis			Multivariable analysis			Univariable analysis			Multivariable analysis		
	HR	95%CI	*p*-value	HR	95%CI	*p*-value	HR	95%CI	*p*-value	HR	95%CI	*p*-value
Female sex	0.808	0.458–1.425	0.461				0.823	0.455–1.488	0.519			
Prior lines of medical therapy >2	2.730	1.689–4.412	<0.001	2.007	1.206–3.342	0.007	2.036	1.199–3.457	0.009			
ECOG PS >0	1.527	1.012–2.305	0.044				1.786	1.135–2.809	0.012			
Squamous	0.579	0.266–1.257	0.167				2.421	0.594–9.866	0.217			
HPV positives	0.579	0.326–1.028	0.062				0.720	0.381–1.358	0.310			
Lung metastases	2.180	1.431–3.319	<0.001				1.367	0.847–2.205	0.201			
Liver metastases	2.489	1.302–4.758	0.006				2.120	1.046–4.297	0.037			
Neutrophils^a^	1.534	0.615–3.829	0.359				3.184	1.265–8.012	0.014			
Leucocytes^a^	1.044	0.318–3.429	0.943				2.707	0.807–9.087	0.107			
Lymophocytes^a^	0.121	0.051–0.289	<0.001	0.218	0.086–0.553	0.001	0.096	0.037–0.252	<0.001	0.144	0.052–0.399	<0.001
dNLR^a^	2.875	1.289–6.414	0.010				6.004	2.453–14.694	<0.001			
Monocytes^a^	1.919	0.566–6.51	0.296				3.433	0.921–12.804	0.066			
Eosinophils^a^	0.702	0.434–1.135	0.149				0.558	0.313–0.996	0.048			
PLT^a^	1.414	0.346–5.777	0.629				3.011	0.694–13.064	0.141			
Albumin	0.920	0.884–0.957	<0.001	0.949	0.911–0.989	0.013	0.903	0.865–0.944	<0.001	0.922	0.879–0.966	<0.001
LDH^a^	1.332	0.797–2.227	0.274				1.212	0.733–2.005	0.454			

The table presents HR, 95% CI, and *p*-values for various clinical and biological variables.

a Results are presented for the log-normalized variable.

95% CI, 95% confidence interval; dNLR, derived neutrophil-to-lymphocyte ratio; ECOG PS, Eastern Cooperative Oncology Group performance status; HPV, human papillomavirus; HR, hazard ratio; LDH, lactate dehydrogenase; *p*-value, probability value; PLT, platelets.

The OS univariable analysis for prognostic factors (Table 2) revealed that prior lines of medical therapy >2 also negatively impacted OS (HR = 2.036, *p* = 0.009), ECOG PS >0 (HR = 1.786, *p* = 0.012), liver metastases (HR = 2.120, *p* = 0.037), and high neutrophils (HR = 3.184, *p* = 0.014) remained a significant negative prognostic factor for OS; lymphocyte count showed a strong protective effect on OS (HR = 0.096, *p* < 0.001), elevated dNLR (HR = 6.004, *p* < 0.001), eosinophils (HR = 0.558, *p* = 0.048), and hypoalbuminemia was predictive of poorer OS (HR = 0.903, *p* < 0.001). In the multivariable analysis for OS, lymphocyte count and albumin levels remain significantly associated with OS (HR = 0.144, *p* < 0.001 and HR = 0.922, *p* < 0.001, respectively).

In conclusion, lymphocytes consistently emerge as a protective factor for both PFS and OS, while hypoalbuminemia predicts poorer outcomes in the multivariable analysis.

No significant differences were observed based on the drug class when analyzing outcomes across different clinical settings, including R/M cases and non-recurrent cases. Similarly, the analysis revealed no notable distinctions in efficacy between patients receiving monotherapy and those undergoing combination therapy (data not shown).

### Safety results

A total of 28 patients (21.5%) experienced at least one of G3/4 TRAEs, with a total of 34 TRAEs (Table 3). The incidence of TRAEs varied across different treatment settings:

- Neoadjuvant: 46.2% (6 out of 13 patients) experienced at least one G3/4 TRAE.
- Adjuvant: no patients in this group experienced G3/4 TRAEs.
- LA: 39.1% (9 out of 23 patients) experienced G3/4 TRAEs.
- R/M: 14.9% (13 out of 87 patients) experienced G3/4 TRAEs.

**Table 3.** Grade 3 or 4 TRAEs: TRAE events are reported using LLT, and each LLT is sorted using the system organ class of the MedDRA classification.

Neoadjuvant (*n* total = 13)	Adjuvant (*n* total = 7)	Locally advanced (*n* total = 23)	Recurrent/metastatic (*n* total = 87)	Total of patients with TRAEs G3 or 4^a^
6 (46.2%)	0 (0%)	9 (39.1%)	13 (14.9%)	28 (21.5%)

G3/G4 TRAEs	Number of events (*n* = 34)
Hematologic parameters	17
Neutrophil count decreased	7
Lymphocyte count decreased	5
Lipase increased	2
Alanine and aspartate aminotransferase increased	2
Creatinine increased	1
Gastrointestinal disorders	5
Oral mucositis	3
Colitis	1
Diarrhea	1
Blood and lymphatic system disorders	3
Febrile neutropenia	3
Metabolism and nutrition disorders	2
Anorexia	1
Hyponatremia	1
Musculoskeletal disorders	1
Arthritis	1
Immune system disorders	1
Anaphylaxis	1
Procedural complications	1
Dermatitis radiation	1
Eye disorders	1
Corneal ulcer	1
Respiratory, thoracic, and mediastinal disorders	1
Dyspnea	1
Cardiac disorders	1
Atrial fibrillation	1
General disorders	1
Fatigue	1

Grade refers to the severity of the AE using the CTCAE 5.0.

a One patient may have had several TRAEs.

CTCAE, common terminology criteria for adverse event; LLT, low-level term; TRAE, treatment-related adverse event.

The most common were impairments in hematologic parameters (17 events), including a decrease in neutrophil count (7 cases) and lymphocyte count (5 cases). Other adverse events included gastrointestinal disorders such as oral mucositis (three cases) and colitis (one case), as well as febrile neutropenia (three cases). Additional TRAEs involved metabolism and nutrition disorders, musculoskeletal issues, immune system reactions, and various other system-specific complications like atrial fibrillation and dyspnea. No treatment-related deaths occurred.

## Discussion

Our landscape study provides a 10-year overview for patients with different subsites of HNC enrolled in phase I trials from a tertiary referral center, demonstrating good tolerability and preliminary efficacy.

Most patients presented with HNSCC, but our cohort also included patients with rare tumors, including salivary gland cancers categorized into adenoid cystic carcinoma (ACC) and non-ACC types (mucoepidermoid and acinic cell carcinomas), treated with innovative drugs of various classes and mechanisms of action. Thanks to this variety of patients, tumors, and treatments, we depict here a representative landscape of the management and clinical outcomes for HNC patients in phase I trials.

The efficacy and safety results of our cohort reassure clinicians considering the proposition of enrollment in phase I trials for their HNC patients. G3/4 TRAEs occurred in 21.5% of patients, without treatment-related deaths. These rates are comparable to, or lower than, those associated with conventional chemotherapy regimens, highlighting the manageable safety profile of phase I agents.^10^

The responses observed in our study align with those seen in HNC patients treated according to standard therapies in the localized/LA setting^16^ while they seem better than standard therapies such as methotrexate or cetuximab administered in previously treated R/M patients.^17,18^

While HNCs are known to have variable prognosis, our efficacy data are comparable to phase I data across other solid tumors. This is consistent with findings from the National Cancer Institute analyses, which report comparable response rates across diverse cancer types in early-stage clinical trials.^19,20^ Recently, the improved survival trends in patients enrolled in phase I trials over recent decades highlight the growing impact of innovative therapies and optimized trial designs.^20^ Moreover, the importance of phase I trials as a critical foundation for advancing cancer treatment has been strongly reaffirmed by the American Society of Clinical Oncology.^21^

As expected, we observed that both ORR and DCR varied significantly across different clinical settings. However, even though the ORR and DCR are significantly lower in the R/M setting, the results obtained in this setting are also reassuring. Indeed, with an ORR of 16.5%, R/M patients seem to present response rates better than those displayed in platinum-resistant HNSCC patients treated with paclitaxel, cetuximab, or methotrexate, according to current European guidelines.^22^ This indicates that even in a challenging metastatic context, phase I trials offer meaningful therapeutic alternatives with potential. Nevertheless, it is important to note that patients enrolled in clinical trials are typically highly selected, often presenting in better overall clinical condition and, moreover, many of whom in our cohort had received only ⩽2 prior lines of therapy. In HPV-negative patients, our data reveal a high DCR and ORR in the earlier treatment settings, particularly in the neoadjuvant and LA stages. These findings suggest that experimental therapies may be particularly effective when administered at an earlier stage. In the R/M setting, HPV-negative patients exhibited lower response rates, with a DCR of 41.8% and an ORR of 16.5%, but clinically meaningful, considering the aggressive nature of HPV-negative tumors. Unlike HPV-positive tumors, which often exhibit a better response to immunotherapy and chemoradiation, HPV-negative tumors are typically associated with more aggressive tumor biology, higher resistance to conventional treatments, and poorer prognosis.^23^ This highlights the urgent need for novel therapeutic strategies tailored to this subset of patients. Despite the challenges associated with HPV-negative disease, our findings are reassuring, particularly in the earlier treatment settings, where the high DCR and ORR suggest a potential role for phase I therapies in improving patient outcomes.

Predictors of poor response to phase I treatments have been well-studied in other cancer types, and our findings largely align with these existing data. Factors such as PS, tumor burden, and previous treatment history remain significant predictors of response in our cohort. A higher ECOG PS score, increased tumor burden, and the number of prior therapy lines demonstrated poorer outcomes, consistent with other studies underscoring their prognostic value in early-phase trials.^19^

The R/M setting was the strongest factor associated with prognostic outcomes, both in univariable and multivariable analyses. The small number of patients and the extreme heterogeneity of our study did not allow us to delve into the importance of the other prognostic factors when analyzed in conjunction with disease settings. Therefore, we conducted an exploratory analysis excluding the disease settings as variables in the univariable and multivariable analyses.

Our analysis revealed that biomarkers such as lymphocyte counts and albuminemia were strongly associated with clinical outcomes, highlighting the critical role of systemic inflammation and nutritional status in shaping patient prognosis. Lymphocytes, as key mediators of the immune response, may enhance tumor surveillance and contribute to better clinical outcomes. A higher lymphocyte count or a favorable dNLR has been associated with improved survival outcomes in HNC and other solid tumors.^24–26^

Conversely, hypoalbuminemia, a marker of malnutrition and systemic inflammation, reflects a compromised physiological state that could limit the patient’s outcome. This is particularly relevant for R/M HNC patients treated with ICIs like nivolumab, where baseline albumin levels ⩾3.5 g/dL were associated with significantly better survival outcomes.^27,28^

Emerging evidence also indicates that enrolling patients in disease-specific trials, as opposed to pan-tumor studies, may enhance outcomes by aligning treatment strategies with tumor-specific characteristics.^19^ However, we are not able to confirm this suggestion in our cohort due to the presence of disease-specific trials, mostly in the localized/LA setting.

One important avenue for future research involves the exploration of new therapeutic classes in HNC and the integration of predictive and pharmacodynamic biomarkers that would not only help refine patient selection but also facilitate hypothesis-driven clinical trials or guide stratification in future trials.^29^

We acknowledge some limitations of our study. First, as a retrospective analysis from a single tertiary referral center, our findings may not be fully generalizable to all HNC patients enrolled in phase I trials. In addition, the heterogeneity of tumors and treatments in our cohort, while reflective of real-world practice, complicates the analysis of specific treatment effects. The small sample size for certain tumor types (e.g., salivary gland cancers) or groups (e.g. HPV-positive HNSCC) limits the ability to draw definitive conclusions for these cancers.

In conclusion, our study provides a comprehensive view of phase I trial outcomes for a large cohort of HNC patients, demonstrating that these trials can offer comparable efficacy to standard treatments with manageable safety profiles. These results support the early inclusion of HNC patients in phase I clinical trials and underscore the potential of experimental therapies in improving outcomes for this challenging cancer type, including HPV-negative disease.

## Conclusion

Our study focuses on patients enrolled in phase I HNC trials, providing reassuring data on the feasibility of offering phase I treatments in terms of both efficacy and safety across various settings. In the R/M setting, particularly in advanced lines where current standard treatments yield modest results, the option of a phase I trial appears to be especially promising. The response rates are higher than those of standard therapies in previously treated patients in the R/M setting, without compromising safety. In conclusion, phase I trials could be considered as a potential therapeutic option for patients with HNC.

## Supplemental Material

sj-docx-1-tam-10.1177_17588359251337244 – Supplemental material for Clinical landscape for patients with head and neck cancers enrolled in phase I trials at a tertiary referral center Supplemental material, sj-docx-1-tam-10.1177_17588359251337244 for Clinical landscape for patients with head and neck cancers enrolled in phase I trials at a tertiary referral center by Daria Maria Filippini, Raphael Sanchez, Etienne Bastien, Nicolas Jacquin, Alexandro Paccapelo, Caroline Nhy, Jerzy Klijanienko, Valentin Calugaru, Anne Chilles, Wahib Ghanem, Guillaume Rougier, Antoine Dubray Vautrin, Nathalie Badois, Maria Lesnik, Olivier Choussy, Marie Paule Sablin, Christophe Le Tourneau and Grégoire Marret in Therapeutic Advances in Medical Oncology

sj-docx-2-tam-10.1177_17588359251337244 – Supplemental material for Clinical landscape for patients with head and neck cancers enrolled in phase I trials at a tertiary referral center Supplemental material, sj-docx-2-tam-10.1177_17588359251337244 for Clinical landscape for patients with head and neck cancers enrolled in phase I trials at a tertiary referral center by Daria Maria Filippini, Raphael Sanchez, Etienne Bastien, Nicolas Jacquin, Alexandro Paccapelo, Caroline Nhy, Jerzy Klijanienko, Valentin Calugaru, Anne Chilles, Wahib Ghanem, Guillaume Rougier, Antoine Dubray Vautrin, Nathalie Badois, Maria Lesnik, Olivier Choussy, Marie Paule Sablin, Christophe Le Tourneau and Grégoire Marret in Therapeutic Advances in Medical Oncology

sj-docx-3-tam-10.1177_17588359251337244 – Supplemental material for Clinical landscape for patients with head and neck cancers enrolled in phase I trials at a tertiary referral center Supplemental material, sj-docx-3-tam-10.1177_17588359251337244 for Clinical landscape for patients with head and neck cancers enrolled in phase I trials at a tertiary referral center by Daria Maria Filippini, Raphael Sanchez, Etienne Bastien, Nicolas Jacquin, Alexandro Paccapelo, Caroline Nhy, Jerzy Klijanienko, Valentin Calugaru, Anne Chilles, Wahib Ghanem, Guillaume Rougier, Antoine Dubray Vautrin, Nathalie Badois, Maria Lesnik, Olivier Choussy, Marie Paule Sablin, Christophe Le Tourneau and Grégoire Marret in Therapeutic Advances in Medical Oncology

## Declarations

**Ethics approval and consent to participate:** Each study received approval from an international review board and a local ethics committee. All patients provided written informed consent for their participation in phase I trials and for the use of their data for research purposes.

**Consent for publication:** The authors are accountable for all aspects of the work in ensuring that questions related to the accuracy or integrity of any part of the work are appropriately investigated and resolved.

**Availability of data and materials:** Aggregated data analyses and the underlying analytic code are available from the authors upon request.
