# Supplementary material for: Clinical landscape for patients with head and neck cancers enrolled in phase I trials at a tertiary referral center
Source: Ther Adv Med Oncol. 2025 Jun 30;17:17588359251337244. doi: 10.1177/17588359251337244 (PMC12209567; doi:10.1177/17588359251337244)
Supplement: sj-docx-1-tam-10.1177_17588359251337244 – Supplemental material for Clinical landscape for patients with head and neck cancers enrolled in phase I trials at a tertiary referral center [file sj-docx-1-tam-10.1177_17588359251337244.docx]

| TRIALS |
| --- |
| NCT05249426 |
| NCT04183166 |
| NCT03260023 |
| NCT03744468 |
| NCT04733027 |
| NCT05462873 |
| NCT05582850 |
| NCT04243499 |
| NCT03526835 |
| NCT05208762 |
| NCT04196283 |
| NCT02997332 |
| NCT01946867 |
| NCT02723955 |
| NCT01772004 |
| NCT03518606 |
| [NCT03170960](http://www.clinicaltrials.gov/show/NCT03170960) |
| NCT01714739 |
| NCT01333085 |
| NCT02822482 |
| NCT02022098 |
| NCT02658890 |
| NCT04089527 |
| [NCT01695005](http://clinicaltrials.gov/show/NCT01695005) |
| NCT02955251 |

**Supplementary table 2:** List of Included Clinical Trials (NCT Numbers).
