# Supplementary material for: Clinical landscape for patients with head and neck cancers enrolled in phase I trials at a tertiary referral center
Source: Ther Adv Med Oncol. 2025 Jun 30;17:17588359251337244. doi: 10.1177/17588359251337244 (PMC12209567; doi:10.1177/17588359251337244)
Supplement: sj-docx-2-tam-10.1177_17588359251337244 – Supplemental material for Clinical landscape for patients with head and neck cancers enrolled in phase I trials at a tertiary referral center [file sj-docx-2-tam-10.1177_17588359251337244.docx]

| Treatment class | Combination | Number of trials | Number of patients |
| --- | --- | --- | --- |
| Immunotherapy | Immunotherapy alone | 13 | 68 |
|  | Immunotherapy and chemotherapy | 2 | 3 |
|  | Immunotherapy and targeted therapy | 1 | 1 |
| Targeted therapy | Targeted therapy alone | 5 | 19 |
|  | Targeted therapy and chemotherapy | 2 | 11 |
| Bispecific antibody | Bispecific antibody alone | 2 | 11 |
| Antibody drug conjugate (ADC) | ADC alone | 1 | 6 |
| Other* | Other alone | 1 | 13 |

*other class was a radiosensitizer

**Supplementary table 1**: Overview of treatment classes and combinations in phase I trials. The table summarizes the number of trials and patients for various therapeutic approaches, including immunotherapy (alone or in combination with chemotherapy or targeted therapy), targeted therapy (alone or with chemotherapy), bispecific antibodies, antibody-drug conjugates (ADCs), and other therapies.
